# Patients With IgA Vasculitis and Kawasaki Disease Show Dysregulated Interferon Signature

**DOI:** 10.1111/1756-185X.70349

**Published:** 2025-07-01

**Authors:** Sevki Erdem Varol, Cisem Cinar, Nihan Burtecene, Sezgin Sahin, Mehmet Yildiz, Kenan Barut, Haluk Cokugras, Sinem Firtina, Ozgur Kasapcopur, Ayca Kiykim

**Affiliations:** ^1^ Department of Pediatrics Cerrahpasa School of Medicine, Istanbul University‐Cerrahpasa Istanbul Türkiye; ^2^ Department of Medical Genetics Cerrahpasa School of Medicine, Istanbul University‐Cerrahpasa Istanbul Türkiye; ^3^ Department of Pediatric Immunology and Allergy Cerrahpasa School of Medicine, Istanbul University‐Cerrahpasa Istanbul Türkiye; ^4^ Department of Pediatric Rheumatology Cerrahpasa School of Medicine, Istanbul University‐Cerrahpasa Istanbul Türkiye

**Keywords:** children, immunoglobulin A vasculitis, interferon signature, Kawasaki disease

## Abstract

**Objective:**

IgA vasculitis (IgAV) and Kawasaki disease (KD) are the most common forms of childhood vasculitis. Although various factors such as viral infections, genetic factors, and environmental factors are involved in the development of both diseases, their pathogenesis remains unclear. The interferon (IFN) signature, which reflects the activation of type I IFN signaling pathways, is a diagnostic and prognostic tool that contributes significantly to the pathogenesis and management of autoimmune diseases. In our study, we aimed to investigate the role of the IFN signature in patients with IgAV and KD.

**Material and Methods:**

Thirty‐two children with IgAV and four patients diagnosed with KD were included in the study. Serum levels of IL‐1, IL‐6, IL‐8, IL‐10, IL‐17, IL‐18, TNF‐α, TNF‐R1, TNF‐R2, and IFN‐gamma were analyzed in the serum samples of all participants, and the expression of IFN‐related genes (*STAT1, IFI27, IFI44, IFI44L, IFIT1*, and *RSAD2*) was assessed in the patients and healthy controls (*n* = 26) to calculate the IFN score by RT‐PCR method.

**Results:**

A significant increase in the expression of three genes (*IFIT1, IFI44,* and *IFI27*) and decreased expression of the other three genes (*RSAD2, STAT1*, and *IFI44L*) was found in patients with IgAV and KD compared to the control group. Significantly higher IFN scores (IFN > 3) were found in patients with gastrointestinal involvement, in patients who required corticosteroid therapy, and in patients who had to be hospitalized. No significant difference in IFN levels was found between patients with and without renal involvement. Significantly higher serum IL‐1 levels were found in patients with gastrointestinal symptoms with IgAV. High IFN scores were found in three out of four patients diagnosed with KD.

**Conclusion:**

A dysregulated type 1 IFN signature was found in patients with IgAV and KD compared to the control group. A significantly increased risk of gastrointestinal involvement required corticosteroid therapy, and hospitalization was observed in patients diagnosed with IgAV who had high IFN levels. This underlines the idea that the IFN score could serve as a crucial prognostic indicator.

## 1 Introduction

Immunoglobulin A vasculitis (IgAV) is the most common vasculitis in childhood and is a disease with multisystem involvement characterized by skin, kidney, gastrointestinal, and joint findings [1, 2]. It is also referred to as IgAV, as IgA deposits can be observed in small vessels [3]. Clinically, IgAV classically presents with palpable purpura without thrombocytopenia and coagulopathy, hematuria due to glomerulonephritis, arthritis, and arthralgia due to joint involvement, abdominal pain, gastrointestinal bleeding, and bowel obstruction due to gastrointestinal involvement [4]. Although most IgAV patients recover with conservative treatment, some patients require the use of corticosteroids [1, 5, 6]. Corticosteroids should be used in the presence of nephritis, orchitis, cerebral vasculitis, pulmonary hemorrhage, and significant gastrointestinal involvement [7, 8, 9, 10].

Kawasaki disease (KD) is an acute febrile clinical illness with vasculitis in medium‐diameter vessels that occurs more frequently in early childhood [1]. The main clinical findings include fever lasting at least 5 days, polymorphous rash, changes in the labial and oral mucosa, bilateral non‐exudative conjunctivitis, unilateral cervical lymphadenopathy, and changes in the limbs [11]. In patients diagnosed with KD, the most important factor for prognosis is the presence of coronary artery involvement and early initiation of treatment during the acute phase of the disease [12, 13]. The main goal of treatment of KD is to suppress acute inflammation, reduce vascular damage, and prevent coronary artery involvement and thrombosis [11]. Initial treatment may include intravenous immunoglobulin, acetylsalicylic acid, corticosteroids in resistant cases, and drugs to suppress tumor necrosis factor (TNF) and interleukin 1 (IL‐1) [11].

The interferon (IFN) signature refers to the dysregulated expression of a series of genes regulated by type I IFNs, a class of cytokines involved in the immune response to viral infections and other pathologic conditions, including autoimmune diseases [14, 15]. Type I IFNs, including IFN‐α and IFN‐β, bind to their receptors on the cell surface and trigger a signaling cascade that leads to the transcription of IFN‐stimulated genes (interferon‐stimulating genes; ISGs). The expression of these ISGs creates the IFN signature, which can be detected in various cell types and tissues [14].

The IFN signature has been observed in the blood and tissue cells of patients with systemic lupus erythematosus (SLE) and other autoimmune diseases, where it serves as a biomarker of active disease and influences treatment selection [14]. In dermatomyositis (DM), the IFN signature correlates with disease activity and has been shown to be more pronounced in patients with anti‐melanoma differentiation gene 5 (MDA5) antibodies [16]. The IFN signature is also associated with the clinical severity of idiopathic inflammatory myopathies and has been proposed as a diagnostic tool [17].

The pathogenesis of IgAV and KD, the most common vasculitis in childhood, is not yet clearly understood. Although the importance of the type I IFN signaling pathway has been demonstrated in many autoinflammatory and autoimmune diseases, there is no study on this topic in IgAV and KD. In this context, we aimed to evaluate the role of the IFN signature measured in children with IgAV and KD in pathogenesis by comparison with healthy individuals.

## 2 Materials and Methods

### 2.1 Patients

Patients who met the diagnostic criteria for IgAV and KD between December 12, 2022 and March 1, 2024 in the Pediatric Rheumatology Department of Istanbul University‐Cerrahpasa Pediatric Rheumatology, General Outpatient Clinic and Pediatric Emergency Outpatient Clinic were included in the study. Patients who did not meet the diagnostic criteria and who were receiving immunosuppressive treatment were excluded from the study.

The control group (*n* = 26) consisted of healthy individuals under the age of 18 who were admitted to the general pediatric outpatient clinic and had no chronic disease, acute infection, or drug use. The patients and healthy controls had a similar gender and age distribution. All patients and healthy controls were informed about the study, and informed consent was obtained from them and their families. The Ethics Committee of Istanbul University‐Cerrahpasa (06.04.2023‐660407) approved the human research, and written informed consent was obtained from all patients and/or their parents.

### 2.2 Laboratory Examinations

#### 2.2.1 Acute Inflammatory Response

Acute inflammatory response including C‐reactive protein (CRP), erythrocyte sedimentation rate [18], white blood cell (WBC) count, absolute neutrophil count [19], platelet count (PLT), hemoglobin concentration (g/dL) were recorded at the time of diagnosis. CRP ≥ 5 mg/L was considered as elevated CRP, WBC count ≥ 12 000/mm^3^ as leukocytosis, ESR ≥ 20 mm/h as elevated ESR, and PLT ≥ 450 000/mm^3^ as thrombocytosis.

#### 2.2.2 Serum Cytokine Analyses

Patient sera were collected at the time of diagnosis prior to treatment, frozen, and stored at 80°C until the measurement. IL‐1, IL‐6, TNF‐α, IL‐17, IL‐8, IL‐18, IFN‐γ, IL‐10, sTNFR1, and sTNFR2 were measured with Bioassay Technology Laboratory (BT‐Lab, Shanghai, China) enzyme‐linked immunoassay [20] kit. Dilution was performed according to the manufacturer's instructions.

#### 2.2.3 Interferon Signature

The peripheral blood samples were collected in ethylenediaminetetraacetic acid (EDTA)‐coated tubes from the patients followed by total RNA isolation with an RNA isolation kit (Invitrogen, USA) prior to treatment. Complementary DNA (cDNA) was synthesized from 1000 ng of total RNA using random primers and a cDNA synthesis kit (Applied Biosystems, USA). Real‐time PCR analysis was performed using the BioRad CF96X system (Applied Biosystems, USA). Primers to all target genes (*STAT1, IFI27, IFI44, IFI44L, IFIT1*, and *RSAD2*) and the housekeeping gene (Beta‐actin) were designed with the Primer3 program, and the primer sequences are available upon request. To calculate the IFN score, the relative expression of targeted genes was calculated by the 2^−∆∆Ct^ method, followed by *Z*‐score‐based standardized IFN score calculation [19] and IFN scores of 3 and more were considered significant.

### 2.3 Statistics

The data were analyzed using the SPSS 29.0.2 program (New York, USA). The compatibility of the variables with the normal distribution was tested using the Kolmogorov–Smirnov test. For variables that did not conform to the normal distribution, the Mann–Whitney *U*‐test was used for the comparison of means between two groups, and the Kruskal–Wallis test for the comparison of means between more than two groups. Spearman's rho correlation coefficient was used to analyze continuous variables, while the chi‐squared test was used for categorical variables. Results are expressed as mean ± standard deviation. Correlations between parameters were reported using Pearson's or Spearman's correlation coefficient depending on the normality of the data. The results were considered statistically significant at *p* < 0.05.

## 3 Results

### 3.1 Clinical Presentation

A total of 32 patients with IgAV and 4 patients with KD were included in our study. Eight IgAV patients were excluded due to insufficient RNA amount, and the IFN signature was evaluated in 24 patients with IgAV and all patients with KD. Of these 28 patients, 16 (57.1%) were female and 12 (42.9%) were male. The mean age of the patients was 7.54 ± 3.79 years. Of the 24 patients diagnosed with IgAV, 14 (58.3%) were female and 10 (41.7%) were male. The mean age of these patients was 8.44 ± 3.23 years. Of the four patients diagnosed with KD, two were male and two were female. The mean age of the patients diagnosed with KD was 2.15 years.

Skin findings were present in all patients with IgAV at presentation. Joint involvement was found in 70.8% (*n* = 17), gastrointestinal involvement in 54.1% (*n* = 13), orchitis in 8% (*n* = 2), and kidney involvement in 16.6% (*n* = 4). When examining the distribution of rashes in the patients, all 24 patients had rashes on the lower extremities. Isolated rash on the lower extremities occurred in 15 patients (62.5%), rash on the upper extremities in addition to the rash on the lower extremities in 4 patients (16.6%), and rash on all extremities and the trunk in 5 patients (20.9%) (Table 1). Evaluation of the patients' classic laboratory findings revealed leukocytosis (WBC ≥ 12 000/mm^3^) in 12 (50%), elevated CRP ((≥ 5 mg/L) L) in 17 (70.8%), and elevated ESR (≥ 20 mm/h) in 12 (50%). Drug treatment (steroids and/or NSAIDs) was initiated in 19 (79.2%) patients with IgAV, 14 (58.3%) of the patients received corticosteroids, 16 (66.6%) received non‐steroidal anti‐inflammatory drugs. Five (20.8%) of the patients were only recommended supportive therapies.

**TABLE 1:** Symptoms seen in patients with IgAV.

	Number (=)	Percentage (%)
Skin		
Purpura	24	100
Subcutaneous edema	5	25
Joint		
Arthralgia only	17	70.8
Arthritis	7	29.2
Gastrointestinal		
Abdominal pain	13	54.1
Nausea and vomiting	11	45.8
Hematochezia	1	4.2
Hematemesis	1	4.2
Melena	3	12.5
Scrotal		
Scrotal edema	2	8
Scrotal pain	2	8.3
Renal		
Hematuria	4	16.7
Proteinuria	1	4.2

All patients with KD fulfilled the diagnostic criteria for classic Kawasaki disease. All patients had elevated CRP and ESR, three patients had thrombocytosis with leukocytosis and one patient lacked both parameters. Echocardiographic examination of the patients revealed dilated coronary arteries in one patient (*Z*‐score between 2 and 2.5) and a giant aneurysm in one patient (*Z*‐score ≥ 10) (Table 2). All patients were treated with IVIG and aspirin as first‐line treatment. Two of the patients were found to be resistant to IVIG treatment. Patients with IVIG resistance also received a second dose of IVIG and high‐dose steroids. As treatment with high‐dose steroids did not respond, anakinra and infliximab were added.

**TABLE 2:** Demographics, clinical, and laboratory data of patients with KD.

	P1	P2	P3	P4
Gender	M	M	F	F
Age (months)	60	4.5	9.7	28.9
Duration of fever (days)	5	12	13	7
Response to IVIG	−	+	+	−
Coronary artery involvement	Dilatation	Giant aneurysm	−	−
Hospital stay length (days)	3	26	14	4
Hemoglobin (g/dL)	5	12	5	6
WBC (/mm^3^)	5000	12 860	34 000	15 600
ANC (/mm^3^)	2600	9990	24 700	9800
PLT (/mm^3^)	248 000	1 115 200	1 482 000	680 000
CRP (mg/L)	20.8	57.2	206.3	32.3
ESR (mm/h)	81	52	35	37
IFN scores	3	4	2	3

Abbreviations: ANC, absolute neutrophil counts; CRP, C‐reactive protein; ESR, Erythrocyte sedimentation rate; F, female; IFN, interferon; IVIG, intravenous immunoglobulin; M, male; P, patient; PLT, platelets; WBC, white blood cells.

### 3.2 Interferon Signature

**FIGURE 1:**
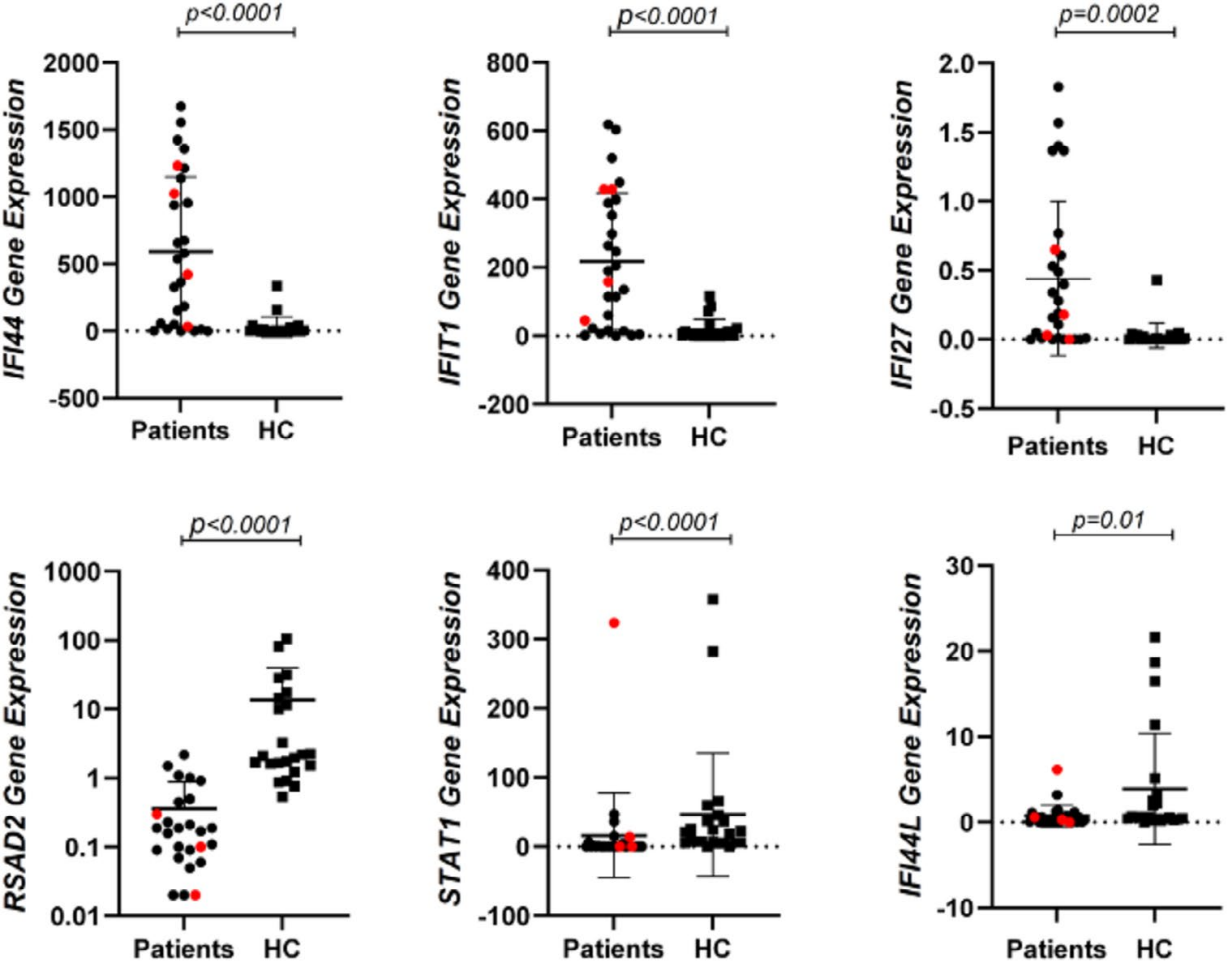
Comparison of the IFN signature between patients and healthy controls. Red dots represent patients with Kawasaki Disease. Black dots represent patients with Immunoglobulin A vasculitis. HC, healthy controls.

In our study, the gene expression of a total of 28 patients (24 with IgAV and 4 with KD) and 26 healthy children was evaluated for IFN signature analysis. Statistical analysis of gene expression data was performed using the Mann–Whitney *U* method. *IFIT1, IFI44*, and *IFI27* gene mRNA expressions were found to be higher in patients (*p* = 0.0002, *p* < 0.0001, and *p* < 0.0001, respectively), whereas *RSAD2, STAT1*, and *IFI44L* gene mRNA expressions were lower in patients (*p* < 0.0001, *p* < 0.0001, and *p* = 0.01, respectively) compared to healthy controls (Figure 1).

When we evaluated the IFN scores, 18 IgAV patients (75%) had an IFN score of 3 or more. In IgAV patients, three (12.5%) patients had an IFN score of 6, one (4%) patient had an IFN score of 5, four (16.6%) patients had an IFN score of 4, 10 (41.6%) patients had an IFN score of 3, and six (25%) patients had an IFN score of 2. There were no IgAV patients with an IFN score of 1. The IFN scores of the Kawasaki patients were calculated as 3, 2, 3, and 4, respectively, for the patients listed in (Table 2).

Symptoms, clinical findings, need for hospitalization, need for corticosteroids, and IFN scores were statistically evaluated. There was no significant difference between the IFN scores of patients with and without joint involvement (*p* = 0.89). IFN scores were significantly higher in patients with gastrointestinal disease overall (*p* = 0.009), including abdominal pain (*p* = 0.09), nausea (*p* = 0.008), and invagination (*p* = 0.021). No significant difference was found with respect to hematemesis, melena, and hematochezia (*p* = 0.789, *p* = 0.313, and *p* = 0.172, respectively). IFN scores showed no significant difference in scrotal involvement, subcutaneous edema, and renal involvement (*p* = 0.82, *p* = 0.46, and *p* = 0.59, respectively). IFN scores were significantly higher in patients requiring corticosteroids (*p* = 0.01).

Patients with a high IFN score had a significantly higher risk of gastrointestinal tract involvement (OR: 4.5) (95% CI: 1.117–18.226) (*p* = 0.034).

Patients with a high IFN score had a significantly higher risk of corticosteroid use (OR: 4.6) (95% CI: 1.091–19.509) (*p* = 0.038).

### 3.3 Comparison of IFN Score With Laboratory Data

Of the patients with IgAV who were included in the study for cytokine analysis, 14 (43%) were male and 18 (57%) were female. The mean age of the patients was 8.28 ± 3.06 years. Of the 45 children in the control group, 24 (53.3%) were male and 21 (47.7%) female. The mean age of the children in the control group was 8.14 ± 3.95 years. No statistically significant difference was found between the children in the patient group and the healthy control group in terms of gender distribution and age.

The laboratory data of patients with IgAV and the IFN score were analyzed using the Spearman correlation analysis method. When the hemoglobin level, WBC count, neutrophil count, lymphocyte count, and PLT were evaluated, no statistically significant correlation was found with the IFN score (*p* = 0.503, *p* = 0.140, *p* = 0.366, *p* = 0.87, and *p* = 0.339, respectively). No statistically significant correlation was found between erythrocyte sedimentation rate, CRP levels, leukocytosis, and IFN score (*p* = 0.872, *p* = 0.530, *p* = 0.108, respectively).

### 3.4 Comparison of the Need for Hospitalization and the Duration of Hospitalization With the IFN Score

Need for hospitalization and length of hospital stay showed higher IFN scores (*p* = 0.005 and *p* = 0.022, respectively). The frequency of hospitalization was significantly higher in patients with a high IFN score (OR: 4.8) (95% CI: 1.190–19.36) (*p* = 0.028).

### 3.5 Evaluation of the IFN Score in Patients With Kawasaki Disease

The IFN score was high in three patients with KD and low in one patient. The duration of fever, IVIG resistance, coronary artery involvement, duration of hospitalization, and IFN scores of the patients are shown in (Table 2).

Among the patients, patient number 2 developed IVIG resistance and had giant aneurysms in his coronary arteries. This patient had an IFN score of 4 (high). Patient number 3 developed IVIG resistance but had an IFN score of 2 (low). In patient number 1, dilation of the coronary arteries was observed and the IFN score was 3 (high). In patient number 4, the coronary artery was not affected and no IVIG resistance was detected. Patient number 4 also had an IFN score of 3 (high).

### 3.6 Evaluation of Cytokine Levels in Patients With IgAV

The levels of IL‐1, IL‐6, IL‐8, IL‐10, IL‐17, IL‐18, TNF‐α, TNF‐R1, TNF‐R2, and IFN‐gamma were determined in serum samples from 31 patients with IgAV. Forty‐five healthy children were included in the control group. In the patient group, 17 patients (54.8%) were female and 14 patients (45.2%) were male. The mean age of the patients was 8.28 ± 3.06 years.

In the control group, 24 (53.3%) children were male and 21 (46.7%) female. The average age of the control group was 8.14 ± 3.95 years. When comparing the patient and control groups in terms of gender and age, no significant difference was found between the two groups.

When comparing the groups, a significant difference was only found in the IL‐6 values (*p* = 0.045). For the other cytokine levels, there was no statistically significant difference between the patient and control groups (Table 3).

**TABLE 3:** Comparison of the cytokine levels of patients with IgAV to healthy controls.

	Patients (*n* = 32)	Healthy controls (*n* = 45)	*p*
Age (years)	8.29 ± 3.06	8.14 ± 3.95	0.86
Gender (female) (*n*, %)	18 (%56.3)	21 (%46.7)	0.55
IL‐1 (ng/mL) median (min–max)	43.2 (3.81–226.2)	55.4 (3.8–222.9)	0.14
IL‐6 (ng/mL) median (min–max)	68.8 (7.05–589.2)	83.1 (14.6–398.3)	**0.045**
IL‐8 (ng/mL) median (min–max)	167.8 (54.5–994.2)	172.2 (50.8–1013.5)	0.98
IL‐10 (pg/mL) median (min–max)	209.4 (10.3–1146.6)	238.1 (10.1–1019.7)	0.71
IL‐17 (ng/mL) median (min–max)	82.9 (6.2–425.5)	87.1 (6.0–361.5)	0.37
IL‐18 (ng/mL) median (min–max)	12.1 (1.5–95.7)	17.7 (1.4–68.5)	0.17
TNF‐α (ng/mL) median (min–max)	164.5 (12.2–1183)	218.4 (11.4–886)	0.34
TNF‐R1 (pg/mL) median (min–max)	7.09 (4,2‐75,5)	9.4 (4.4–52.9)	0.07
TNF‐R2 (ng/mL) median (min–max)	687.3 (338.7–2814.3)	659 (250.4–3401.8)	0.85
IFN‐ɣ (ng/mL) median (min–max)	41.1 (24.3‐41.1)	54.1 (25.2–270.4)	0.07

*Note:* Significant values are given in **bold**.

### 3.7 Comparison of Serum Cytokine Levels According to Clinical Findings in Patients With IgAV

The cytokine levels did not differ regarding arthritis in IgAV compared to healthy controls. However, in patients with arthralgia the serum levels of TNF‐R2 and IL‐8 were significantly higher (*p* = 0.012 and *p* = 0.056, respectively). Patients with abdominal pain had higher serum IL‐1 and TNF‐α levels (*p* = 0.004 and *p* = 0.017, respectively).

The presence of GI findings when evaluated together revealed higher serum IL‐1 levels (*p* = 0.004), this was also true for vomiting (*p* = 0.028). In patients with melena, the serum IL‐18 level was higher (*p* = 0.025), and in patients with intussusception serum levels of TNF‐R1 and IFN‐ɣ were significantly higher compared to patients without intussusception (*p* = 0.033 and *p* = 0.033, respectively).

Cytokine levels did not differ with regard to renal and/or scrotal involvement and diarrhea.

Serum levels of TNF‐R1 and IFN were significantly lower in patients with isolated lower extremity rash (*p* = 0.038 and *p* = 0.038, respectively). A significant increase in the levels of IL‐1, TNF‐α, TNF‐R1, and IFN‐ɣ was found in patients receiving corticosteroids (*p* = 0.013, *p* = 0.009, *p* = 0.045, and *p* = 0.045, respectively).

There was no correlation between the IFN score and the serum cytokine levels (Spearman's rank correlation coefficient).

## 4 Discussion

The etiopathogenesis of IgAV and KD, the most common childhood vasculitides, is not yet fully understood. This study aimed to investigate the type 1 IFN signature in IgAV and KD, as well as the relationship between cytokine levels, clinical findings, and IFN scores in patients with IgAV. To achieve this, we examined the expression of six genes (*IFI27*, *IFI44*, *IFI44L, IFIT1, RSAD2*, and *STAT1*) associated with the type 1 IFN signaling pathway. Our analysis revealed a significant increase in the expression of *IFIT1, IFI44*, and *IFI27* in patients with IgAV and KD compared to the control group. In contrast, the expression of *RSAD2, STAT1*, and *IFI44L* was decreased relative to controls (Figure 1).

Type 1 interferonopathies, which were first defined as an independent group of autoimmune diseases in 2011, now include dermatomyositis, systemic lupus erythematosus, and primary Sjogren's syndrome [15, 21, 22]. In addition, increased expression of type 1 IFN‐related genes has been shown to be associated with disease activity in patients with rheumatoid arthritis [23]. A study by Batten et al. examined the type 1 IFN signature in ANCA‐associated vasculitis and found that there was no increase in gene or protein expression of the type 1 IFN signaling pathway between ANCA‐associated vasculitis and the healthy control group [24]. In our study, a significant increase in the expression of *IFIT1, IFI44*, and *IFI27* was found when the patient group with IgAV and KD was compared with healthy controls. This finding suggests that the type 1 IFN signaling pathway may play a role in disease pathogenesis. The fact that the expression of the *RSAD2, STAT1*, and *IFI44L* genes was reduced in the patient group compared to the control group indicates that there is a disturbance in the regulation of the IFN signaling pathway in the patient group.

Patients' IFN signature scores were calculated using the standardized IFN score calculation method based on the *Z*‐score [25]. When comparing the IFN scores of patients diagnosed with IgAV with and without gastrointestinal tract involvement, the IFN score was significantly higher in patients with gastrointestinal tract involvement (*p* = 0.009). The risk of gastrointestinal tract involvement was significantly higher (OR: 4.5) (95% CI: 1.117–18.226) in patients with high IFN scores (*p* = 0.034). A high IFN score in patients with IgAV could be a biomarker for gastrointestinal involvement.

The IFN scores did not differ in IgAV patients with regard to renal involvement (*p* = 0.59). This result suggests that the IFN score may not be an effective indicator of renal involvement in patients with IgAV. However, the limited sample size may have affected the power of our results. Therefore, future studies with larger samples should be conducted to determine whether the IFN score can be a reliable biomarker for predicting renal involvement in IgAV patients. A longitudinal study to assess renal involvement should be warranted.

In IgAV patients, those requiring corticosteroid treatment had significantly higher IFN scores than those who did not (*p* = 0.01). Additionally, patients with high IFN scores had a significantly increased risk of needing corticosteroid treatment (OR: 4.6, 95% CI: 1.091–19.509, *p* = 0.038). High IFN scores may serve as an important prognostic factor for corticosteroid treatment necessity.

IgAV patients with gastrointestinal and renal involvement may require hospitalization. Those needing hospitalization had significantly higher IFN scores (*p* = 0.005). A high IFN score was associated with a greater risk of hospitalization (OR: 4.8, 95% CI: 1.190–19.36, *p* = 0.028) and longer hospital stays. These findings highlight the IFN score as a crucial prognostic marker.

This study aimed to investigate the IFN signature in KD patients. However, due to insufficient mRNA isolation, this was not possible in 6 of the 10 patients. The small sample size limits our assessment, but 3 of the 4 analyzed patients had high IFN scores. Future studies should reevaluate the IFN score in KD with a larger patient group.

There are few studies showing an increase in cytokines in patients with IgAV. In a study by Tae‐Sun Ha, patients diagnosed with IgAV nephritis were compared with patients without nephritis, and it was found that serum and urinary TNF‐α levels were significantly higher in patients with IgAV nephritis [26]. In a study of 20 IgAV patients conducted in Turkey, elevated levels of TNF‐α, IL‐1, and IL‐6 were found in skin biopsies [27]. In addition, a gene polymorphism of IL‐18, a proinflammatory cytokine, has been reported to play a role in the development of IgAV [28, 29]. In a study conducted by Novak et al. to evaluate anti‐ *Helicobacter pylori* antibodies in patients with IgAV, it was shown that patients' TNF‐α levels were elevated compared to the healthy control group [30]. We found IL‐6 levels significantly lower in IgAV patients than in the control group (*p* = 0.045), suggesting a role for vascular inflammation in disease development. However, this contradicts the expected pro‐inflammatory role of IL‐6. No significant differences were observed in other cytokine levels between the patient and control groups.

There are studies that show an increased risk of IgAV in patients with familial Mediterranean fever (FMF) [31, 32]. In a study conducted on 80 IgAV patients in Turkey, a heterozygous mutation in the *MEFV* gene was found in 34% of patients [32]. In addition, patients carrying mutations in the *MEFV* gene were found to be younger and more likely to have arthritis and edema compared to those without mutations [32]. This study also showed that CRP and ESR values were higher in patients with *MEFV* mutations [32]. FMF is closely associated with dysregulation of interleukin‐1 (IL‐1), particularly IL‐1β. Mutations in the *MEFV* gene, which encodes the pyrin protein, lead to uncontrolled IL‐1β release, which is one of the main factors of inflammation in FMF [33]. IL‐1 levels were significantly higher in IgAV patients with gastrointestinal involvement than in those without (*p* = 0.0004). This finding highlights a potential link between IgAV and FMF. While studies suggest that IgAV patients with MEFV mutations experience more arthritis and edema [32], no research has examined IL‐1 levels in this group. Future studies should investigate both MEFV gene mutations and IL‐1 levels in IgAV patients.

When comparing the cytokine levels of patients with and without joint symptoms (arthritis and/or arthralgia) in patients diagnosed with IgAV, it was found that TNF‐R2 and IL‐8 levels were statistically significantly higher in patients with joint symptoms (*p* = 0.012 and *p* = 0.056, respectively). There are studies in the literature showing that serum TNF receptors are elevated in patients with rheumatoid arthritis and juvenile rheumatoid arthritis [34, 35]. There is no autoimmune or autoinflammatory disease directly associated with IL‐8. Elevated serum TNF‐R2 and IL‐8 levels as a result of immune dysregulation induced in patients with IgAV may be responsible for the joint symptoms experienced by patients.

When comparing patients with IgAV with and without melena, serum IL‐18 levels were statistically significantly higher in patients with melena (*p* = 0.025). As mentioned above, studies have shown that a gene polymorphism of the proinflammatory cytokine IL‐18 is associated with the development of IgAV. In our study, patients with melena had high serum IL‐18 levels.

In the majority (68.8%) of IgAV patients in our study, the rash was limited to the lower extremities, while in some patients it spread to the upper extremities and trunk. No statistically significant results were found when comparing patients with and without isolated lower extremity rash in terms of hospitalization, corticosteroid requirements, renal involvement, and gastrointestinal involvement. In a large IgAV cohort conducted by Ekinci et al., subcutaneous edema on extremities was found to be significantly less frequent in patients with severe GI involvement than without [36]. When analyzing patients' cytokine levels with respect to rash distribution, TNF‐R1 and IFN levels were significantly lower in patients with isolated lower extremity rash (*p* = 0.038 and *p* = 0.038, respectively). The significant increase in serum TNF‐R1 and IFN‐ɣ levels in patients whose rash spread to the trunk and upper extremities may be a sign of increased inflammation in the patients.

Our analysis found no significant difference in cytokine levels between IgAV patients with and without renal involvement. In contrast, Tae‐Sun Ha's study reported significantly higher serum and urine TNF‐α levels in IgAV nephritis patients [26]. We did not observe a similar increase in serum TNF‐α levels in our nephritis group (*n* = 5), though our small sample size limits the generalizability of these findings.

In patients who required corticosteroids, IL‐1, TNF‐α, TNF‐R1, and IFN‐ɣ levels were significantly higher (*p* = 0.013, *p* = 0.009, *p* = 0.045, and *p* = 0.045, respectively), which may indicate increased inflammation in patients requiring corticosteroid treatment.

This work is important as it is the first study to examine the type 1 IFN signature in IgAV and KD, two childhood vasculitis. The results of this study will be important for future studies in expanded patient populations.

In our study, the IFN signature in patients with IgAV was evaluated, revealing dysregulation of the IFN signaling pathway. This finding suggests that the IFN pathway may play a crucial role in the pathogenesis of IgAV and raises the possibility that JAK inhibitors targeting this pathway could represent a novel therapeutic strategy. Notably, previous case reports have documented clinical improvement in patients with IgAV following JAK inhibitor treatment, further supporting this hypothesis [37, 38]. However, the precise mechanisms underlying IFN pathway dysregulation in IgAV remain unclear. Future studies, including larger‐scale clinical trials, are warranted to confirm these findings and assess the therapeutic efficacy and safety of JAK inhibitors in this patient population.

The cross‐sectional nature of our study, the lack of longitudinal data, and the limited number of KD patients are the limitations of this study. However, this is the first study to present the IFN signature in children with IgAV.

In conclusion, the IFN signature may provide an indication of gastrointestinal involvement and disease severity in the acute phase in patients with IgAV. Gastrointestinal involvement, corticosteroid requirement, hospitalization rate, and length of hospital stay are higher in IgAV patients with high IFN levels. As renal involvement may occur during the course of the disease, prospective studies are needed to demonstrate the efficacy of the IFN signature as a prognostic biomarker for renal involvement during follow‐up.

## Author Contributions

S.E.V., S.F., C.C., N.B., A.K., S.S., M.Y., K.B., H.C., O.K. conceived and planned the experiments. C.C., S.F., N.B. carried out the experiments. S.S., M.Y., K.B., H.C., O.K. contributed to sample preparation. S.E.V., S.F., C.C., N.B., A.K., S.S., M.Y., K.B., H.C., O.K. contributed to the interpretation of the results. S.E.V. and A.K. took the lead in writing the manuscript. All authors provided critical feedback and helped shape the research, analysis, and manuscript.

## Conflicts of Interest

The authors declare no conflicts of interest.

## Data Availability

The data that support the findings of this study are available on request from the corresponding author. The data are not publicly available due to privacy or ethical restrictions.
